# The effects of grazing on daily caloric intake and dietary quality

**DOI:** 10.1186/s12966-021-01226-4

**Published:** 2021-12-18

**Authors:** Eliana Zeballos, Carolyn Chelius

**Affiliations:** 1 USDA Economic Research Service, Food Economics Division, Washington, D.C., USA; 2 USDA Economic Research Service, Food Economics Division, Kansas City, MO USA

**Keywords:** NHANES, Caloric intake, Grazing, Dietary quality, Healthy Eating Index, HEI-2015, Eating patterns

## Abstract

**Background:**

The duration and frequency of eating occasions has been identified as a factor contributing to poor dietary quality among U.S. adults. The objective of this study is to examine whether grazing, defined as eating more than three times a day, affects total daily caloric intake and dietary quality measured by the 2015 Healthy Eating Index (HEI-2015).

**Methods:**

We used a multivariate individual fixed-effects model to compare the caloric intake and dietary quality of individuals who grazed on 1 day but not another. This allowed us to control for differences in individual food intake and diet quality preferences among study participants. We use the National Health and Nutrition Examination Survey (NHANES), 2007-2018, and include data for adults aged 18 years or older who reported 2 days of dietary intake and were not pregnant or lactating (*n* = 27,775).

**Results:**

Grazing increased total daily caloric intake by 205 cal and increased the daily HEI score by 0.59 points. Grazing increased HEI component scores for total fruit, whole fruit, and refined grains, and decreased HEI component scores for saturated fats. Morning grazing increased total daily caloric intake by 159 cal and increased the daily HEI score by 0.87 points — primarily by increasing component scores for total fruit, whole fruit, whole grains, total dairy, seafood and plant proteins, and sodium. Evening grazing increased daily caloric intake by 76 cal and decreased the daily HEI score by 0.41 points — primarily by decreasing the component scores for total fruit, whole grains, fatty acids, and saturated fats. Evening grazing increased HEI component scores for sodium and refined grains.

**Conclusions:**

Grazing increases daily caloric intake and can decrease dietary quality (particularly when grazing in the evening).

## Introduction

Adult dietary quality is of significant public health concern, as poor dietary quality is associated with a range of health conditions, including increased risk of heart disease, stroke, Type II diabetes, and several types of cancer [1]. Poor dietary quality contributes to the prevalence of overweight and obesity, which has substantial medical and economic costs. Globally, 53% of the adult population is overweight or obese, and the annual medical cost of obesity is an estimated $990 billion [29, 30]. Given these statistics, there is a clear incentive to identify factors that contribute to a healthy diet and limit excess weight gain among adults. We focus in particular on U.S. adults, as the prevalence of overweight and obesity among this population is 72%, but our analysis also has relevance for other populations.

The duration and frequency of eating occasions have become of increasing interest as a potential factor in mitigating the public health issue of dietary-related disease. Americans consume more of their calories as snacks, and not as part of the typical dietary pattern of three eating occasions per day [28]. 2020 marks the first year the Dietary Guidelines for America (DGA) Scientific Advisory Committee examined the relationship between duration and frequency of eating events and dietary quality and health outcomes [27]. However, the DGA Committee concluded there was not enough research on the relationship between eating occasion frequency and health to generate recommendations on eating occasion frequency in the final Dietary Guidelines. The DGA Scientific Advisory Committee stated “this question is important to pursue in future cycles [of the DGAs]” and asserted there is a “critical need for additional research” on the topic [27].

A dietary pattern at the center of this research is “grazing” — a pattern characterized by a high number of eating occasions per day, though the exact definition is contested [3, 11]. In psychology studies, grazing has been called “the unstructured, repetitive eating of small amounts of food,” “constant overeating,” “picking and nibbling,” and “chaotic/ unstructured eating” [11]. In the medical community, grazing has been characterized as eating “not in response to hunger and satiety cues” — with “compulsive and non-compulsive” components [3].

These different definitions prevent the accurate measurement of “grazing” and render it difficult to compare data across studies. Several studies have analyzed the impact of frequency and duration of eating occasions on dietary quality — a few of which have specifically looked at “grazing.” Eicher-Miller et al. [6] show adhering to a dietary pattern of three evenly spaced, proportionally equivalent eating occasions throughout the day is associated with higher dietary quality compared to eating five or more times during the day. Leech et al. [16] show grazing, as opposed to following a “conventional” or “late lunch” dietary pattern, is associated with lower dietary quality and increased consumption of discretionary calories, in particular from snacks. Other studies find opposite results. Keast, Nicklas, and O’Neil [14] find snacking frequency and an increased percentage of calories from snacks (as opposed to “meals”) decreases the likelihood of obesity among adolescents. Hamermesh [10] shows grazing is associated with lower body mass index and better self-reported health.

While there is no consensus on the definition or impact of grazing, the topic has widespread implications across multiple disciplines, including psychology, nutrition, medicine, and policy. This study adds to the literature by shedding light on this understudied, inconclusive topic. The objective of this study is to analyze the impact of grazing on caloric intake and dietary quality. Grazing in this study is defined as participating in more than three eating occasions per day, though we challenge this definition and explore its nuances. We use a multivariate individual fixed effects model to analyze differences in caloric intake and dietary quality among U.S. adults who graze 1 day and adhere to a three-eating-occasion dietary pattern another. We use the 2 days of dietary intake data from the 2007-18 National Health and Examination Survey (NHANES). This novel statistical method allows us to account for individual food intake and diet quality preferences, and more accurately assess the impact of grazing.

## Methods

### The National Health and Nutrition Examination Survey and the dietary intake component

We use the National Health and Nutrition Examination Survey (NHANES), which is a continuous survey of the noninstitutionalized U.S. civilian population conducted annually by the National Center for Health Statistics within the Center for Disease Control and Prevention. The survey collects dietary recall data from individuals as well as demographic, medical, and physiological information to assess Americans’ health and nutrition [2, 12, 33].

The NHANES dietary recall component — called the “What we eat in America (WWEIA) survey” — is administered over 2 days, typically 3 to 10 days apart. Day 1 of survey administration is conducted in person, at the NHANES’s Mobile Examination Center; Day 2 of survey administration is conducted over the phone. Both days of survey administration require individuals to recount what they ate and at what time, for the past 24 h (from midnight to midnight). NHANES uses the USDA Automated Multiple-Pass Method to maximize the accuracy of the recall process [19, 22]. NHANES is considered the best data for analyzing population-level dietary intake [26]; however, the data still suffer from underreporting— especially for day 2 [19, 24].

In addition to the WWEIA survey, NHANES collects demographic data from individuals, such as ethnicity and socioeconomic status, and anthropometric measurements and laboratory test data during the in-person interview.

### Study sample

Our study uses the six most recent years of publicly available NHANES data, covering 2007-18. We include participants aged 18 years or older (*n* = 32,426) who consumed more than 9 kcal per day (*n* = 32,423), reported 2 days of dietary intake (*n* = 28,191), and who were not pregnant (*n* = 232) or lactating (*n* = 184). Our final sample size was 27,775.

### Identifying meals and measuring intake

As stated in the introduction, there is no consistent definition of grazing. Grazing has been described in the literature as “picking and nibbling,” indicating grazers consume few calories per day, but also as “constant overeating,” implying grazers consume more than the necessary number of calories per day [11]. One consistent feature of grazing, however, is a high number of eating occasions per day [3, 11].

Key to the definition of grazing is the definition of an “eating occasion.” We defined an eating occasion as any occasion in which food or drink was consumed and calories ingested were greater than 9 kcal. Calorie thresholds to define an eating occasion are common: Khanna et al. [13], Leech et al. [15], Zeballos and Todd [31], St-Onge et al. [25], and Murakami and Livingstone [20] use a 50-kcal threshold to define an eating occasion. However, Hamermesh [10] and Conceição et al. [3] do not use a calorie threshold to define an eating occasion. We use a threshold of > 9 kcal in this study, as we were interested in capturing small (and potentially healthy) eating occasions — a handful of berries is less than 50 cal, as is a mini granola bar, for example. However, we wanted to eliminate eating occasions consisting of low to zero-calorie foods and beverages, such as water. Using a > 9 kcal threshold, the eliminated eating occasions in our study totaled about 3.22% of our sample and consisted mainly of water, coffee, and tea. We recognized our choice of calorie threshold had the potential to influence our results, so we controlled for average calories per eating occasion in our regression and performed robustness checks with three different thresholds for calorie count per eating occasion: > 0 kcal, > 49 kcal, and > 199 kcal. We elaborate on these results in the discussion.

After establishing a definition of an “eating occasion,” we then defined “grazing.” We conceptualized “grazing” as the continual ingestion of calories throughout the day, but needed to determine how to analyze the data empirically. Critical to this determination is the distinction between “meals” and “snacks.” Like grazing, there is no standard definition of a “meal” or “snack,” and multiple approaches have been used in the literature. One method of distinction is to use a self-identification approach (e.g., using dietary recall surveys where participants self-identify whether their eating event was “breakfast,” or a “snack” [20, 25]). Another is to use the time of day (e.g., any eating occasion between 6 am and 10 am is considered “breakfast)” [20, 25]. A third method is to use its percent contribution to total daily caloric intake (e.g,. any eating occasion constituting > 15% of total daily calories is considered a “meal”) [20, 25]. There are other less common methods, such as latent class analysis approach, which can be used to define meal patterns [15].

Many of these studies [20, 25] did not specifically look at grazing when analyzing the impact of meal and snack frequency on dietary quality, making it challenging to use their definitions. Indeed, a few specifically looked at the *difference* between the impact of “meal” and “snack” frequency on dietary quality [17, 20, 21]. Leech et al. [15], on the other hand, looked specifically at grazing, by using latent class analysis to uncover dietary patterns among study participants. Leech et al. [15] found the “grazing” dietary pattern was characterized by high meal and snack frequency, with “frequent, but no distinct peaks in probability of meal consumption.” We used these findings as a guidepost, as they were in keeping with our concept of grazing as the continual ingestion of calories throughout the day. Leech et al.’s results [15] suggest distinguishing “meals” and “snacks” by time of day or percent contribution to caloric intake may not be relevant to “grazing.” We decided there was no real reason to distinguish between “meals” and “snacks,” and we defined grazing as participating in more than three eating occasions per day. There is precedent for this definition, as Conceição et al. [3] use the same criterion. We performed robustness checks by recalculating our results when defining grazing as recording more than four eating occasions per day and recording more than five eating occasions per day. We elaborate on the results and the implications of our methodological choices in the discussion.

In addition to deciding on a caloric threshold for defining an eating occasion, we also had to determine whether to include drinks in our analysis. Khanna et al. [13], Eicher-Miller et al. [6], Hamermesh [10], and Leech et al. [15] include drinks in the definition of grazing; Conceição et al. [3] exclude drinks, and Eicher-Miller et al. [6] do not specify whether drinks are included. We decided to include drinks, in keeping with our conceptualization of grazing as “the continual ingestion of calories throughout the day.” We viewed drinks such milk and soda, as important to our analysis.

From here on, we use “eating occasion,” to refer to any occasion in which food is consumed, whether that be considered a “meal” or “snack.” We counted the total number of eating occasions on both day 1 and day 2 for each participant, and grazers were identified as individuals who recorded more than three eating occasions on one or both days of dietary intake data collection. We also compared the total caloric intake and dietary quality of morning grazers to that of evening grazers. Individuals were defined as morning grazers if they reported more than two eating occasions between 3:00 a.m. and 2:59 p.m. Individuals were defined as evening grazers if they reported more than one eating occasion between 3:00 p.m. and 2:59 am [31, 32]. Our analytic sample for the main analysis included 27,775 respondents.

The total calories consumed per day was calculated as the sum of all calories reported. We calculated the ratio of calories from food-away-from-home (FAFH) establishments as the sum of calories from FAFH divided by the total calories consumed during the day. We calculated the average calories per eating occasion by dividing the total number of calories consumed per day by the total number of eating occasions per day. We calculated the average time between eating occasions by calculating the average number of minutes between the start of each eating occasion. The time between the first and last eating occasion was calculated by looking at the time difference between the last eating occasion of the day and the first eating occasion of the day. We also controlled for whether the data collection was on day 2, since research shows there is a higher incidence of underreporting calories for the second day of NHANES data collection [18], and controlled for whether the day of data collection fell on weekend, as research has shown daily caloric intake is statistically significantly higher on weekend days as compared to weekdays [9].

### Assessing nutritional quality

To measure nutritional quality, we appended the WWEIA survey with the Food and Nutrient Database for Dietary Studies, which allowed us to analyze the nutritional composition of individuals’ diets using the 2015 Healthy Eating Index score (HEI-2015). The HEI score measures individuals’ dietary compliance with the USDA *Dietary Guidelines for Americans* (DGAs), and it is considered a reliable measure of dietary quality in the nutrition community [23]. The DGAs, along with the HEI score, are updated every 5 years by the USDA Center for Nutrition for Policy and Promotion, to reflect the most recent consensus in nutrition science.

Our study uses the HEI-2015 — the latest iteration of the index, which aligns with the recommendations in the 2015-2020 DGAs. As with the previous DGAs, the 2015-2020 edition emphasizes consuming a variety of food groups, focusing on nutrient density, and maintaining a diet within calorie needs [4]. New to the 2015 DGAs is a specific recommendation to limit the intake of added sugars to less than 10% of total caloric intake. The HEI-2015 includes nine adequacy components (i.e., food groups or nutrients that people should consume at least a certain amount of every day), and four moderation components (nutrients that should be limited). Adequacy components include total fruit, whole fruit, total vegetables, greens and beans, whole grains, total dairy, total protein foods, seafood and plant proteins, and fatty acids [4]. Moderation components include refined grains, sodium, added sugars, and saturated fats [4]. Consumption of the adequacy components contributes to a higher HEI score overall, and consumption of the moderation components contributes to a lower HEI score overall. In addition to using the total HEI score to examine the nutritional quality of our study participants’ diets, we analyzed the HEI component scores, to assess measures of specific nutrients.

### Statistical methods

We present summary statistics for day 1, day 2, and both days of data collection (Table 1). We calculated the proportion of grazers, morning grazers, and evening grazers, and calculated average total calories, average total HEI-2015 score, and average HEI-2015 component scores (Table 1). Day 1 and day 2 means were compared using t-tests. We then compared demographic characteristics among those who grazed both days, those who grazed 1 day, and those who did not graze either day (Table 2). We used t-tests to compare if the mean of one subgroup was different from the mean of another subgroup (e.g., those who grazed both days compared to non-grazers). In Table 1, we placed a superscript “a, b, or c” next to the mean if a particular subgroup is statistically significantly different from the mean of another subgroup (“a” for comparisons between those who grazed both days and those who grazed on 1 day, “b” between those who grazed both days and those who did not graze, and “c” between those who grazed on 1 day and those who did not graze). Unless otherwise indicated, all differences we discuss in the text between subgroups of study participants are significant at the 95% level of confidence (i.e., *p* < 0.05).

**Table 1:** Means and standard errors of demographic characteristics by eating pattern over 2 days, adults age 18 and older

	All		Grazers both days			Grazers 1 day			Non grazers		
	Mean	SE	Mean		SE	Mean		SE	Mean		SE
Male	48.6	0.407	46.8	a,b	0.539	51.3	a	0.928	52.7	b	1.250
Age (years)	46.8	0.267	48.1	a,b	0.289	45.1	a,c	0.377	43.6	b,c	0.464
Married or living with partner	52.8	0.787	56.7	a,b	0.853	47.7	a,c	1.027	42.4	b,c	1.399
Hispanic	14.5	0.982	13.4	a,b	0.915	16.4	a	1.198	16.1	b	1.401
Non-Hispanic, White	66.2	1.403	70.1	a,b	1.340	61.2	a,c	1.643	56.2	b,c	1.893
Non-Hispanic, Black	11.5	0.768	8.6	a,b	0.589	14.9	a,c	1.040	19.5	b,c	1.409
Less than high school	14.4	0.596	12.1	a,b	0.596	17.4	a,c	0.789	20.4	b,c	1.113
High school graduate or GED	22.2	0.546	20.9	a,b	0.646	23.1	a,c	0.865	27.5	b,c	1.223
Some college or AA degree	30.4	0.560	30.0	a	0.723	32.5	a,c	0.893	27.7	c	1.221
College graduate or above	29.5	0.962	34.4	a,b	1.121	22.3	a,c	0.970	18.5	b,c	1.256
Observations	27,775		16,343			7994			3438		

Notes: The superscripts “a, b, or c” are placed if a subgroup is statistically significantly different from the mean of another subgroup (“a” for comparisons between those who grazed both days and those who grazed on 1 day, “b” between those who grazed both days and those who did not graze, and “c” between those who grazed on 1 day and those who did not graze) (*p* < 0.05). Standard errors account for complex survey design. NHANES dietary intake day 2 weights (wtdr2d) were used to compute nationally representative estimates. Statistical significance of differences across groups are evaluated using a t-test stastic

Source: Authors’ calculations using data from the 2007-18 National Health and Nutrition Examination Survey (NHANES) and https://epi.grants.cancer.gov/hei/developing.html#2015c

**Table 2:** Summary of intake measures from NHANES, day 1 and day 2, adults age 18 and older

	Day 1		Day 2	
	Mean	SE	Mean	SE
Grazers	**78.9**	0.513	**72.8**	0.589
Morning grazers	**48.4**	0.579	**45.4**	0.652
Evening grazers	**71.9**	0.466	**64.1**	0.486
Total energy content	**2141.4**	8.495	**2027.7**	10.848
HEI-2015 (max = 100)	**50.9**	0.240	**51.6**	0.219
Adequacy components (max):				
Total vegetables (5)	3.1	0.020	3.1	0.019
Greens and beans (5)	1.526	0.027	1.511	0.024
Total fruits (5)	**2.1**	0.028	**2.2**	0.029
Whole fruits (5)	**2.1**	0.033	**2.2**	0.031
Whole grains (10)	**2.5**	0.048	**2.8**	0.043
Dairy (10)	5.1	0.041	5.1	0.046
Total protein foods (5)	4.2	0.014	4.2	0.014
Seafood and plant proteins (5)	2.3	0.029	2.3	0.027
Fatty acids (10)	5.0	0.045	5.0	0.038
Moderation components (max):				
Na (10)	**4.3**	0.033	**4.1**	0.039
Refined grains (10)	6.2	0.038	6.2	0.045
Saturated fats (10)	5.9	0.039	5.9	0.044
Added sugars (10)	**6.7**	0.048	**6.9**	0.044
Observations	27,775		27,775	

Notes: HEI, Healthy Eating Index. In parenthesis, the maximum number of points each element contributes to the HEI-2015. In boldface, if the difference between the mean of day 1 and the mean of day 2 is statistically different from zero (*p* < 0.05). Standard errors account for complex survey design. NHANES dietary intake day 2 weights (wtdr2d) were used to compute nationally representative estimates. Statistical significance of differences across days are evaluated using a t-test stastic

Source: Authors’ calculations using data from the 2007-18 National Health and Nutrition Examination Survey (NHANES) and https://epi.grants.cancer.gov/hei/developing.html#2015c

To estimate the effect of grazing on total daily caloric intake, total daily HEI-2015 score, and the 13 component scores, we used a first difference model — a fixed-effects model with two observations for each individual. We calculated the difference between variables over the 2 days (day 2 – day 1) which removes all individual characteristics that do not change between the 2 days (e.g., demographic variables and unobserved consumption preferences). This leaves only the variables that vary between the 2 days, allowing the model to estimate how changes in day-to-day eating patterns affect the dependent variables. The fixed effect assumption is that by removing any of the omitted variation due to time-constant factors and by controlling for time-variant factors, the error term is uncorrelated with the independent variables [8]. The constant term reflects differences in the means across the 2 days.

The model to estimate the effect of grazing on daily caloric intake, HEI-2015 score, and HEI-2015 component scores included an indicator for grazing, a ratio for calories prepared away-from-home (vs. at-home), the average number of calories per eating occasion, the average time between eating occasions, the time between the first and last eating occasion of the day, an indicator for whether the intake day was day 2, and an indicator for whether the intake day fell on a weekend. We performed the same regression for both “morning” and “evening” grazers and conducted two robustness checks: one where we changed the eating occasion threshold we used to define grazing, and another where we changed the calorie threshold we used to define an eating occasion.

**Fig. 1:**
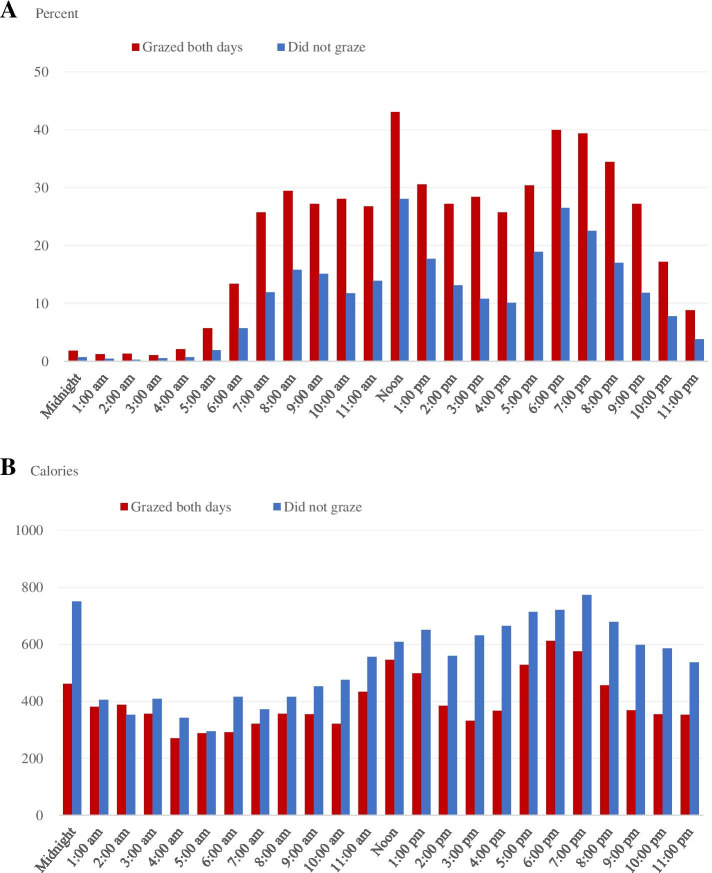
Percentage of Americans who engaged in eating and average calories when engaged in eating, by the time of day, on an average day in 2007-18, age 18 and older, and those who grazed 1 day and did not the other day. **A** Percent engaged in eating. **B** Average calories when engaged in eating. Notes: Weighted means reported. NHANES dietary intake day 2 weights (wtdr2d) were used to compute nationally representative estimates. Source: Authors’ calculations using data from the 2007-18 National Health and Nutrition Examination Survey (NHANES)

**Fig. 2:**
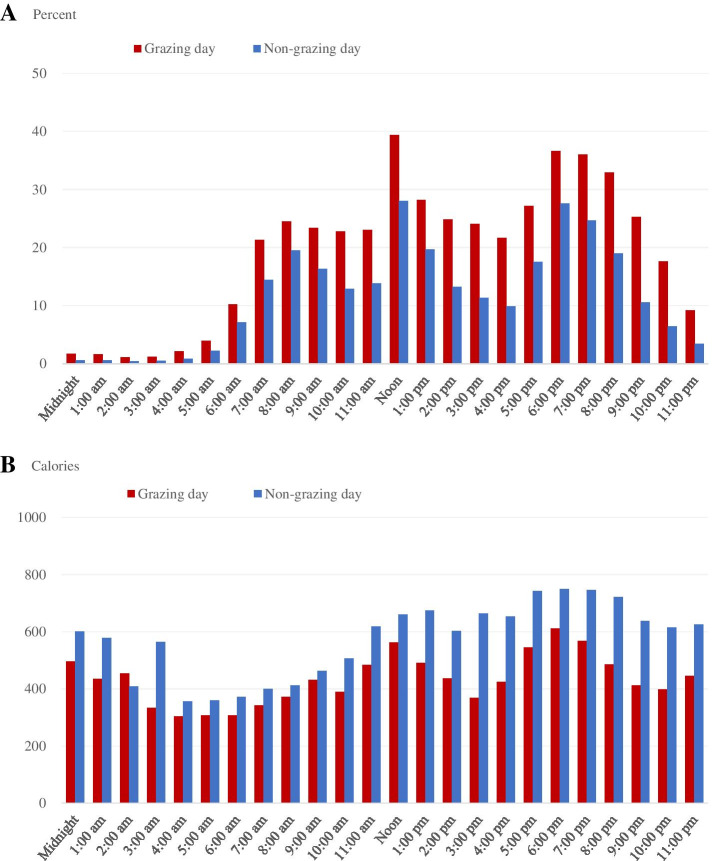
Percentage of Americans who engaged in eating and average calories when engaged in eating, by the time of day, on an average day in 2007-18, age 18 and older and those who grazed both days and those who did not graze. **A** Percent engaged in eating. **B** Average calories when engaged in eating. Notes: Weighted means reported. NHANES dietary intake day 2 weights (wtdr2d) were used to compute nationally representative estimates. Source: Authors’ calculations using data from the 2007-18 National Health and Nutrition Examination Survey (NHANES)

Finally, we graphically explored the relationship between grazing, caloric consumption, and time of day. We calculated the percentage of individuals in our sample (i.e., those who grazed 1 day and did not graze the other day) that ate at each hour of the day, broken down by the day they grazed and the day they did not graze (Fig. 1a). We also calculated the average number of calories consumed by individuals in our sample engaged in eating at each hour of the day, broken down by the day they grazed and the day they did not graze (Fig. 1b). We conducted the same graphical analysis for individuals who grazed both days, and individuals who did not graze on either day (Fig. 2). We calculated averages using individuals’ recorded caloric intake and time of eating occasion.

All analyses were conducted using the survey-related commands in the statistical software package Stata version 14.2 with the intake day 2 weights applied and standard errors adjusted to account for the complex survey design.

## Results

### Sample demographics

Our sample included 27,775 participants. The mean age of our study participants was 47 years old, and 48.6% of participants were male. 66.2% identified as non-Hispanic white, 11.5% as non-Hispanic black, and 14.5% identified as Hispanic. 52.8% of our sample indicated they were married or living with partner. 14.4% recorded their highest level of education as less than high school, 22.2% as high school graduate or GED, 30.4% as some college or AA degree, and 29.5% as college graduate or above.

### Comparing grazers on both days, to grazers on 1 day, and to non-grazers

Individuals who grazed on 1 day were different from those who did not graze either day. Individuals who grazed on 1 day were on average older, more educated, more likely to be married, and more likely to be non-Hispanic White than those who did not graze (*p* < 0.05) (Table 2). Individuals who grazed on 1 day were also different from those who grazed both days. Individuals who grazed on 1 day were on average younger, less educated, less likely to be married, and more likely to be Hispanic or non-Hispanic Black than those who grazed on both days (*p* < 0.05).

### Summary of intake

On average, 78.9% of all individuals grazed the first day, and 72.8% grazed the second day. Most grazing happened in the evening. When looking at day 1, 71.9% grazed in the evening compared to 48.4% who grazed in the morning. Day 2 follows a similar pattern: 45.4% of American adults grazed in the morning and 64.1 grazed in the evening (Table 1). By design, the results from the first difference model consider only those who grazed on 1 day and did not graze on the other day (28.8% of our sample). Similarly, when analyzing how morning or evening grazing affects caloric intake and dietary quality, results from the first difference model considers only those who grazed in the morning on 1 day but not in the morning on the other day (35.8%), or those who grazed in the evening on 1 day but not in the evening of the other day (36.0%). Thirteen percent grazed in the morning and the evening on 1 day but did not graze at all on the other day.

Mean caloric intake on day 2 was lower than on day 1 (2028 kcal versus 2141 kcal, *p* < 0.05). Mean HEI-2015 score was lower on day 1 (50.9) than on day 2 (51.6) with total fruit, whole fruit, whole grains, and added sugars component scores also lower on day 1 (*p* < 0.05). The score for sodium was higher on day 1 (*p* < 0.05).

### Effects of grazing

Adults consumed 205 more calories when grazing (*p* < 0.01), 159 more calories when grazing in the morning (*p* < 0.01), and 76 more calories when grazing in the evening (*p* < 0.01) (Table 3). The average daily caloric intake on the weekend was greater, while the mean reported calorie intake on day 2 was lower, and the more calories that were consumed from food-away-from-home (FAFH) establishments, the more calories were consumed during the day (*p* < 0.05). Individuals who consumed a higher number of calories per eating occasion on average consumed more total calories per day (*p* < 0.01), and individuals who consumed meals more spaced apart consumed fewer calories per day (*p* < 0.01). Individuals who had a larger time difference between their last eating occasion of the day and their first eating occasion of the day (i.e. a greater total range of eating hours) consumed more calories per day.

**Table 3:** Coefficients and standard errors from first difference regressions of total daily caloric intake on grazing and other intake characteristics

	(1)		(2)		(3)	
	Total daily caloric intake		Total daily caloric intake morning grazing		Total daily caloric intake evening grazing	
	Coefficient	SE	Coefficient	SE	Coefficient	SE
Grazing	204.56***	8.726	159.27***	6.082	76.24***	6.377
Day 2	−40.26***	3.398	−43.38***	3.389	−39.37***	3.432
Weekend	42.10***	4.660	48.94***	4.654	40.66***	4.700
Average calorie per eating occasion	2.98***	0.013	2.98***	0.013	2.96***	0.013
Average time between eating occasions	−3.20***	0.037	−3.42***	0.032	−3.61***	0.032
Ratio of FAFH calories	0.20**	0.086	0.16*	0.086	0.19**	0.087
Time between first and last eating occasion	1.94***	0.019	0.16*	0.016	2.11***	0.017
Constant	−191.20***	14.087	− 166.30***	13.626	−106.90***	13.547
Observations	27,775		27,775		27,775	
R2	0.71		0.71		0.71	

Notes: FAFH, food-away-from-home. *** *p* < 0.01, ** *p* < 0.05, * *p* < 0.1. Standard errors account for complex sampling design. NHANES dietary intake day 2 weights (wtdr2d) were used to compute nationally representative estimates. Coefficients and standard errors are estimated from first difference regressions (fixed effect model)

Source: Authors’ calculations using data from the 2007-18 National Health and Nutrition Examination Survey (NHANES)

Grazing increased diet quality as measured by the daily HEI-2015, by about 0.59 points (*p* < 0.05) (about 1.2% relative to the mean). Grazing in the morning had a larger effect (0.87 points, *p* < 0.01) while grazing at night decreased the HEI score by 0.41 points (*p* < 0.05). Grazing decreased the component scores for saturated fats (*p* < 0.05), and increased component scores for refined grains (*p* < 0.10), total fruit (*p* < 0.05), and whole fruit (*p* < 0.01) (Table 4). Most of the positive changes in the different component scores were driven by morning grazing. Morning grazing increased the scores for total dairy (*p* < 0.05), total fruit, whole fruit, whole grains, seafood and plant proteins, and sodium (*p* < 0.01). However, morning grazing decreased the score for total vegetables (*p* < 0.05). Evening grazing decreased the component scores for total fruit (*p* < 0.05), whole grains, fatty acids, and saturated fats (*p* < 0.01), and increased the scores for sodium and refined grains (*p* < 0.01).

**Table 4:** Summary of results of the effect of grazing on total HEI-2015 and the 13 component scores, first difference regression

	(1)		(2)		(3)	
	Grazing		Morning grazing		Evening grazing	
	Coefficient	SE	Coefficient	SE	Coefficient	SE
Daily HEI-2015 score	0.59**	0.24	0.87***	0.17	−0.41**	0.17
Adequacy components, scores:						
Total vegetables	0.01	0.03	−0.06**	0.02	−0.03	0.03
Greens and beans	0.03	0.05	0.04	0.03	−0.04	0.03
Total fruit	0.09**	0.04	0.18***	0.03	−0.07**	0.03
Whole fruit	0.17***	0.04	0.20***	0.03	0.00	0.03
Whole grains	0.04	0.06	0.12***	0.04	−0.21***	0.05
Total dairy	0.08	0.07	0.11**	0.05	0.00	0.05
Total protein foods	0.04	0.03	−0.01	0.02	−0.02	0.02
Seafood and plant proteins	0.05	0.05	0.11***	0.03	0.02	0.03
Fatty acids	0.03	0.08	0.02	0.05	−0.18***	0.06
Moderation components, scores:						
Na	0.11	0.07	0.13***	0.05	0.29***	0.05
Refined grains	0.13*	0.07	0.00	0.05	0.14***	0.05
Saturated fats	−0.16**	0.07	0.05	0.05	−0.27***	0.05
Added sugars	−0.04	0.06	−0.03	0.04	−0.04	0.04

Notes: HEI, Healthy Eating Index. *** *p* < 0.01, ** *p* < 0.05, * *p* < 0.1. Standard errors account for complex survey design. NHANES dietary intake day 2 weights (wtdr2d) were used to compute nationally representative estimates. Additional controls include whether the data collection was on the second day, whether the day of data collection fell on weekend, average calorie per eating occasion, the average time between eating occasions, the ratio of FAFH eating occasions, and the time between the first and last eating occasion. Observations: 27,743. Coefficients and standard errors are estimated from first difference regressions (fixed effect model)

Source: Authors’ estimations using data from the 2007-18 National Health and Nutrition Examination Survey (NHANES)

When we defined grazing as participating in more than four eating occasions per day and more than five eating occasions per day, we found a larger effect on total daily caloric intake. When we defined grazing as participating in over four eating occasions per day, the average total calories increased by 250 kcal (*p* < 0.01); when we defined grazing as participating in more than five eating occasions per day, the average total calories increased by 260 kcal (*p* < 0.01). The remaining coefficients in our model remained significant and in the same direction as when we defined grazing as participating in over three eating occasions per day (Table 5). The effect on the daily HEI-2015 is lower when grazing is defined as participating in over four eating occasions a day (0.47, *p* < 0.05) and insignificant when grazing is defined as participating in over five eating occasions per day (not shown).

**Table 5:** Coefficients and standard errors from first difference regressions of total daily calorie intake on grazing and other intake characteristics, with different exclusion thresholds

	(1)		(2)	
	Total daily caloric intake > 4 eating occasions per day		Total daily caloric intake > 5 eating occasions per day	
	Coefficient	SE	Coefficient	SE
Grazing	249.80***	6.946	260.37***	7.232
Day 2	−35.03***	3.360	−33.52***	3.362
Weekend	43.37***	4.600	43.11***	4.600
Average calorie per eating occasion	3.00***	0.012	2.98***	0.012
Average time between eating occasions	−3.11***	0.034	− 3.35***	0.031
Ratio of FAFH eating occasions	0.22***	0.085	0.21**	0.085
Time between first and last eating occasions	1.91***	0.017	2.01***	0.016
Constant	− 164.16***	13.282	− 117.05***	13.108
Observations	27,775		27,775	
R2	0.72		0.72	

Notes: FAFH, food-away-from-home. *** *p* < 0.01, ** *p* < 0.05, * *p* < 0.1. Standard errors account for complex sampling design. NHANES dietary intake day 2 weights (wtdr2d) were used to compute nationally representative estimates. Coefficients and standard errors are estimated from first difference regressions (fixed effect model)

Source: Authors’ calculations using data from the 2007-18 National Health and Nutrition Examination Survey (NHANES)

When we estimated the effect of grazing when eliminating the calorie exclusion criteria, excluding eating occasions with less than 50 cal (about 9.5% of all reported eating occasions), and excluding eating occasions with less than 200 cal (about 33.5% of all reported eating occasions); we found a larger effect on total daily caloric intake (210 kcal, 225 kcal, and 357 kcal, respectively; *p* < 0.01) (Table 6).

**Table 6:** Coefficients and standard errors from first difference regressions of total daily calorie intake on grazing and other intake characteristics, with different exclusion thresholds

	(1)		(2)		(3)	
	Total daily caloric intake no restrictions		Total daily caloric intake 50 cal threshold		Total daily caloric intake 200 cal threshold	
	Coefficient	SE	Coefficient	SE	Coefficient	SE
Grazing	210.20***	8.953	225.39***	8.361	356.70***	8.187
Day 2	−41.67***	3.403	−39.58***	3.397	−36.39***	3.597
Weekend	42.40***	4.670	43.01***	4.667	50.43***	4.951
Average calorie per eating occasion	3.05***	0.013	2.83***	0.012	2.03***	0.011
Average time between eating occasions	−3.29***	0.038	− 2.92***	0.036	− 1.63***	0.027
Ratio of FAFH eating occasions	0.22**	0.086	0.20**	0.086	0.58***	0.092
Time between first and last eating occasion	1.93***	0.019	1.95***	0.019	2.19***	0.018
Constant	−200.74***	14.453	− 192.93***	13.361	−250.39***	12.077
Observations	27,778		27,764		27,476	
R2	0.71		0.71		0.67	

Notes: FAFH, food-away-from-home. *** *p* < 0.01, ** *p* < 0.05, * *p* < 0.1. Standard errors account for complex sampling design. NHANES dietary intake day 2 weights (wtdr2d) were used to compute nationally representative estimates. Coefficients and standard errors are estimated from first difference regressions (fixed effect model)

Source: Authors’ calculations using data from the 2007-18 National Health and Nutrition Examination Survey (NHANES)

Finally, we graphically depicted the relationship between grazing, caloric consumption, and time of day. Fig. 1a displays the percentage of individuals in our sample (i.e., those who grazed 1 day and did not graze the other day) that ate at each hour of the day, broken down by “grazing day” and “non-grazing” day. “Grazing day” refers to the day out of the 2 days of dietary intake in which the individual grazed; “non-grazing day” refers to the day out of the 2 days of dietary intake in which the individual did not graze.

Figure 1a shows the percentage of individuals in our sample that ate at each hour of the day is higher on “grazing day” than on “non-grazing day” for all hours of the day (*p* < 0.05). As depicted in Fig. 1a, the eating pattern of the “non-grazing day” followed a trimodal, or three-peaked, distribution, with the percentage of people engaging in eating peaking between 8 and 8:59 a.m., then again between noon and 12:59 p.m., and then once more between 6 and 6:59 p.m. The first of these three peaks, however, was smaller than the second and third, with just 19.5% of our sample engaging in eating at the 8 a.m. peak, compared to 28% at the noon hour and 27.6% at the 6 p.m. hour. The eating pattern of the “grazing day” was different: it followed a binominal, or two-picked, distribution, with the percentage of people engaging in eating peaking between noon and 12:59 p.m. and then again between 6 and 6:59 p.m. However, on average, 23% of our sample ate at each hour from 7:00 a.m. until noon on the grazing-day, compared to 15.4% on the non-grazing day.

Figure 1b shows the average number of calories individuals in our sample consumed at each hour of the day, broken down by “grazing day” and “non-grazing” day. The average number of calories was higher on the “non-grazing day” than on the “grazing day” for all hours of the day except for the 2:00 a.m. hour (*p* < 0.05). The figure shows the highest peaks of caloric consumption on “non-grazing day” occur between noon and 1:59 p.m. (667 kcal, on average) and between 5 and 8:59 p.m. (740 kcal, on average). This makes sense, as these times are associated with the consumption of lunch and dinner. Additional peaks occur at midnight, 1 a.m., and 3 a.m.; however, less than 2 % of individuals ate at these hours. On the “grazing day,” the highest peaks of caloric consumption are still at typical times associated with a three-eating-occasion per day pattern, but the values are not as high as on “non-grazing day” (i.e., 562 kcal at the noon hour and 612 kcal a the 6 p.m. hour). The difference between the average calories consumed at each hour of the day on “non-grazing day” and “grazing day” was larger during the hours of 12:00 p.m. - 12:00 a.m.

We replicated this graphical analysis for individuals who grazed both days and individuals who did not graze on either day (Fig. 2). We found our results were consistent with the analysis of individuals who grazed 1 day and not the other. A higher percentage of individuals who grazed on both days ate at all hours of the day than did the percentage of individuals who did not graze on either day (Fig. 2a), and non-grazers consumed more calories at all hours of the day than did grazers, except for at the 2:00 a.m. hour (Fig. 2b). The largest difference between non-grazers’ and grazers’ average caloric consumption occurred during the afternoon and evening hours (Fig. 2b).

## Discussion

In this study, we examined whether grazing — participating in a dietary pattern characterized by the continual ingestion of calories throughout the day — influences total daily caloric intake and dietary quality. Our study compares the dietary intake of individuals who graze 1 day and not another, rather than comparing individuals who graze to individuals who do not graze. The latter approach — which is predominantly used in the literature — is problematic, as it can yield biased results. For example, it is possible grazers engage in other healthy behaviors, such as eating more fruits and vegetables, which could lead to lower daily caloric intake and an increased HEI score. It is also possible grazers engage in unhealthy behaviors, such as eating foods high in sugar or saturated fat, which would lead to a higher daily caloric intake and a lower HEI score. Our fixed-effects model allows us to control for observed and unobserved individual characteristics such as these and isolate the effect of grazing on daily caloric intake and diet quality.

Grazing was associated with increased daily caloric intake and a higher overall HEI score. However, when separating grazers into “morning grazers” and “evening grazers” we observed the positive effect on HEI score to be largely driven by morning grazing. Morning grazing is associated with higher component scores for total fruit, whole fruit, whole grains, total dairy, and seafood and plant proteins — all of which increase the overall HEI score. Specifically, morning grazers consume 1.6 times more fruit, in cups-equivalent, than do non-morning grazers. Conversely, evening grazing is associated with decreased component scores for total fruit, whole grains, and fatty acids — all of which decrease the overall HEI score. Morning grazing may increase the total HEI score because certain healthy foods are associated with the morning, such as fruits, nuts, and whole-grain products [5]. Similarly, foods high in nutrients that lower the HEI score, such as candy and potato chips, are often consumed in the evening. Research substantiates this pattern: individuals who eat late at night are more likely to consume foods high in sugar and fat [7].

Our results align with the literature on the dietary quality of food-away-from-home, as well as the caloric intake reported during the days of NHANES data collection. Individuals in our study consumed more calories on average, and more calories of lower nutritional value, on average, when eating food prepared away-from-home, as compared to eating food prepared at-home. Individuals in our sample also consumed more calories, on average, on day 1 of NHANES data collection as compared to day 2. Both findings are consistent with the literature, adding credibility to our results ([18].)

While our study is strengthened by using a fixed-effects approach over 2 days of dietary intake data, the lack of additional days of data per individual is a limitation. It is possible we analyze food intake data that are not characteristic of respondents’ typical eating patterns (e.g., perhaps the individual had been traveling or celebrating a holiday during the day of dietary recall). It is also possible some varying, unobservable disturbances may still be correlated with the explanatory variables. For example, an individual may have worked from home 1 day and had easy access to a refrigerator, but another day worked in a chemistry lab and had strict limitations on when he or she could eat. We also cannot determine whether the choice to graze was intentional. An individual may have chosen to graze as a deliberate way to consume less food, or an individual may not have even realized he or she was grazing. Our study analyzes the effect of grazing regardless of the individual’s intentions to graze.

Another potential limitation of our study is our inclusion of shift workers. Several studies analyzing the impact of meal patterns on dietary quality have intentionally eliminated shift workers from the study sample, as research has shown shift work to be associated with higher risk of metabolic syndrome, and calories consumed late at night to be of lower quality than calories consumed at other times of day [15]. We considered eliminating shift workers from our analysis; however, NHANES does not have data on participants’ work schedules for all years of data in our analysis, nor does NHANES have data on participants’ work schedules on the actual days of data collection. We conducted a sensitivity analysis (not shown) where we conducted our analysis using only rounds of NHANES data where shift work data were available, and found grazing still had a positive impact on daily caloric consumption as well as morning and evening caloric consumption. This finding aligns with Murakami et al. [20], which showed meal frequency, snack frequency, and dietary quality were similar among night/evening/rotating workers and regular day/non-workers. We chose to include shift workers in our study and not limit our sample to a smaller number of observations, but we acknowledge our results could still be biased. Future research could study the impact of grazing on dietary quality, and differentiate between shift-workers and non-shift workers.

Another potential limitation of our study is there is no standard definition of “grazing,” “meal,” “snack,” or even “eating occasion.” To explore alternative definitions, we conducted robustness checks where we changed our calorie threshold to define an eating occasion from > 9 kcal to > 0 kcal, > 49 kcal, and > 199 kcal. Our results were consistent in all three scenarios. We also conducted robustness checks where we changed the definition of grazing as participating in more than four eating occasions per day and participating in more than five eating occasions per day. Again, our results were consistent.

Our decision not to distinguish “meals” from “snacks” in our analysis also could have impacted our results. Leech et al., [17] found increased snacking frequency is associated with lower dietary quality, whereas increased meal frequency is not. Murakami and Livingstone [21], however, find both meal frequency and snack frequency are associated with overweight and obesity. While we chose not to distinguish between meals and snacks, our results were similar to Leech et al. [16] — which distinguishes between meals and snacks, and finds grazing is associated with increased caloric consumption. Additional research is recommended to further explore the relationship between “meals,” “snacks,” “grazing,” and dietary quality.

Another aspect of defining grazing that could potentially have impacted our results were the time cut-offs for defining “morning” and “evening” grazers. We explored this time component graphically, in Figs. 1 and 2, but did not define grazing based on time of day, as other studies have done ([6, 13]. Additional studies could explore these nuances in defining grazing and the resulting impact on caloric intake and dietary quality.

A final area for further research is the cultural variation in dietary patterns. We use NHANES data, which limits our analysis to U.S. consumers, but dietary-related disease is a global issue, and there is a need for research on the effect of grazing on the diets of populations outside of the U.S., using data other than NHANES. Exploring the impact of grazing on the dietary quality of subgroups within populations is also important. Our study controls for individual fixed effects (such as religion or race), but we do not explicitly analyze the effect culture might have on grazing, and thus on dietary quality. This is significant, as our point of comparison to grazing — consuming three, evenly spaced eating occasions per day — is not globally the norm.

## Conclusions

In aggregate, grazing — as defined as participating in more than three eating occasions per day — increases daily caloric intake and dietary quality as measured by the HEI-2015. However, grazing in the morning — defined as consuming more than two eating occasions between 3:00 a.m. and 2:59 p.m. — increases overall HEI score, specifically by increasing HEI component scores for total fruit, whole fruit, and seafood and plant proteins. Grazing does not appear to be an effective calorie reduction strategy, in aggregate, or even an effective strategy at improving most individual components of dietary quality. However, morning grazing could contribute to higher fruit and protein intake. It is important to note, though, that morning grazers still increased their overall caloric intake compared to non-grazers, even though they consumed foods that improved their HEI score. It appears grazing does not replace consuming lunch, and dinner (calorie-dense eating occasions) at conventional times; however, these eating occasions have fewer calories, on average, than when individuals do not graze.

Our results contribute to the literature on eating patterns and dietary quality, and specifically shed light on grazing, where there is currently no conclusion on its impact on caloric intake and dietary quality. In addition to providing analysis to answer this question, we raised critical points in how to define grazing.

Our study has demonstrated holistic, nuanced advice may be necessary when communicating recommendations concerning grazing to the public. Recommendations that emphasize the types of foods that are eaten when grazing, the time of day, and the nutrients within each food could be helpful. It may be possible to increase dietary quality via grazing, but it is entirely dependent on the foods consumed, and the nutrients in need of increasing or decreasing. For example, our findings suggest an individual looking for ways to increase whole fruit intake may benefit from morning grazing, whereas an individual looking to decrease refined grain consumption may benefit from avoiding evening grazing.

While these findings may be useful for forming future recommendations concerning “grazing,” there is still a need for additional research to inform dietary policy recommendations. Consistent definitions for “eating event,” “snack,” “meal,” and “grazing” are needed, as well as additional geographically and culturally diverse research. 

## Data Availability

The datasets generated and/or analysed during the current study are available on the NHANES website, https://www.cdc.gov/nchs/nhanes/index.htm.

## Abbreviations

HEI: Healthy Eating Index
NHANES: National Health and Nutrition Examination Survey
DGA: Dietary Guidelines for America
USDA: United States Department of Agriculture
WWEIA: What we eat in America
FAFH: Food-away-from-home
